# Calico

**Published:** 1871-08

**Authors:** 


### CALICO

Was the name given to a cloth made of cotton, and sent for sale throughout the world from the town of Calicut in India. The finer kinds of it are called "muslin," manufactured at a place bearing a name somewhat similar. Fifty years ago the amount of calico woven was about three million yards, but it now amounts to about five hundred millions of yards every year; it is healthier for wear than silk.

A hundred and fifty years ago a law was passed in England, with severe penalties, against any person who wore or traded in "painted, stained, or dyed calico," because means had been found in India to dye calicoes,—an unknown art in Great Britain; the colored or printed material was so popular, that the plain white kind which the English made and traded in had no sale, and caused incalculable suffering among the working-people in calico manufactories; not even a chair could be covered, or a window curtain made of the prohibited material; but the times have changed, and muslins are printed with any pattern almost as easy as newspapers.

